# Kirk’s Hand-Book of Physiology

**Published:** 1885-07

**Authors:** 


KIRKE'S HAND-BOOK OF PHYSIOLOGY. By W. Morrant Baker, F. R. C. S., Surgeon to St. Bartholomew's Hospital and Consulting Surgeon to the Evelina Hospital for Sick Children; Lecturer on Physiology at St. Bartholomew's Hospital, and late Member of the Board of Examiners of the Royal College of Surgeons of England; and Vincent Dormer, M. D., London, Demonstrator of Physiology at St. Bartholomew's Hospital. Eleventh Edition, with nearly five hundred Illustrations. In two Volumes. New York: William Wood & Co. 1885.

These two volumes comprise the February and March numbers of "Wood's Library of Standard Medical Authors" for the present year. The fact that the work has gone through eleven editions is the very best evidence of its merit. Many important new facts and observations which are fairly established have been added, and a large number of new illustrations have also been added, making the work a most valuable one for students.

The publishers are to be congratulated upon the handsome appearance of the "Library," and the profession ought to encourage them in their efforts to give them standard medical literature at a nominal cost.

